# Supporting Healthcare Workers on the Frontline of Conflicts: Comment on "Human Resources for Health in Conflict Affected Settings: A Scoping Review of Primary Peer Reviewed Publications 2016–2022"

**DOI:** 10.34172/ijhpm.9457

**Published:** 2025-11-12

**Authors:** Andrew MY Lim, Kevin KC Hung, Chi Shing Wong, Chunlan Guo, Joyce TK Ching, Emily YY Chan, Colin A. Graham

**Affiliations:** ^1^Emergency Department, Prince of Wales Hospital, Hong Kong SAR, China.; ^2^Collaborating Centre for Oxford University and CUHK for Disaster and Medical Humanitarian Response (CCOUC), Chinese University of Hong Kong, Hong Kong SAR, China.; ^3^Accident and Emergency Medicine Academic Unit, Chinese University of Hong Kong, Hong Kong SAR, China.; ^4^Department of Geography and Resource Management, Chinese University of Hong Kong, Hong Kong SAR, China.

**Keywords:** Attacks on Healthcare, International Humanitarian Law, Violence Against Healthcare

## Abstract

The rising trend of conflict-related deaths, the repeated attacks on healthcare facilities, and violations of the International Humanitarian Law (IHL) seen in recent conflicts are not only worrying but also deserve our collective attention and efforts. The scoping review on human resources for health in conflict-affected settings (CAS) from 2016 to 2022 by Onvlee and colleagues provides a timely reminder of the need for immediate relief and the longer-term support needed to provide healthcare in these extremely challenging settings. In this article, we reflect and elaborate on some of the key findings from Onvlee et al, including attacks on healthcare, the performance of healthcare workers (HCWs), and long-term strategic planning for the health workforce in CAS.

 Onvlee and colleagues’ scoping review on human resources for health in conflict-affected settings (CAS) from 2016-2022 provides a timely reminder of the need for immediate relief and the longer-term support needed to provide effective healthcare in these extremely challenging settings.^1^ According to the Peace Research Institute Oslo (2025), state-based armed conflicts have increased, and the period between 2021 and 2024 has been identified as the most violent since the end of the Cold War.^2^

 Bendavid et al estimated in the Lancet series on *Women’s and Children’s Health in Conflict Settings* that between 1995 and 2015, over 10 million deaths in children less than five years old globally were attributable to conflict, while women living near high-intensity conflict zones had a mortality rate three times higher than those in peaceful regions.^3^ The health workforce is one of the essential building blocks in health systems, and workforce planning is essential to establish a functioning health workforce. In this article, we reflect and elaborate on some of the key findings from Onvlee et al.

## Attacks on Healthcare

 Following the adoption of the World Health Assembly Resolution 65.20 in 2012, the World Health Organization (WHO) established the Surveillance System for Attacks on Health Care to systematically collect data on assaults against healthcare in fragile, conflict-affected, and vulnerable settings. The most recent figures are alarming. In the first 10 months of 2025 alone, there were 1122 documented attacks, resulting in 1727 deaths and 1007 injuries.^4^ Incidents occurred across 16 countries and territories, with the highest concentration in the occupied Palestinian territories, Ukraine, the Democratic Republic of the Congo, Sudan, Syria, and Myanmar. Other affected regions included Burkina Faso, Haiti, Iran, Israel, Lebanon, Libya, South Sudan, Russia, and Yemen. Most incidents had an impact on health facilities (n = 764) or personnel (n = 518), while patients (n = 398), transport (n = 357), supplies (n = 265), and warehouses (n = 117) were impacted to a lesser extent. Heavy weapons were the most frequently employed means of attack, followed by obstruction, psychological violence, and individual weapons.

 It is particularly concerning to note that as of November 4, 2025, the death toll so far in 2025 from attacks on healthcare (n = 1727) has already surpassed the annual totals recorded in 2024 (n = 944) and 2023 (n = 762) (Figure). This reflects an increasing frequency and lethality of attacks on healthcare. The Director-General of the WHO, Dr. Tedros Adhanom Ghebreyesus, has repeatedly condemned such attacks, calling “for the protection and respect of health workers in humanitarian settings and beyond.”^5^

**Figure:**
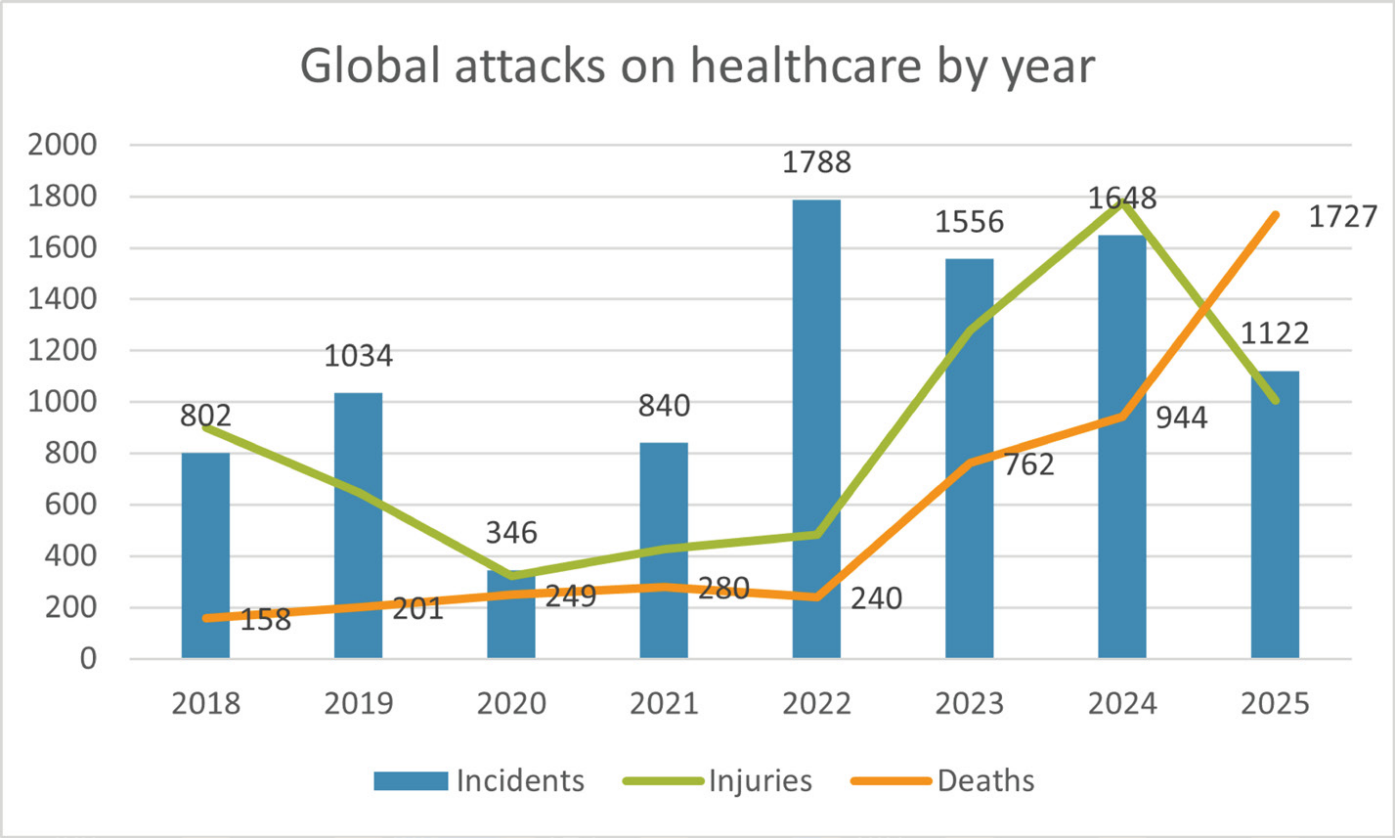
Global Attacks on Healthcare by Year (According to the World Health Organization Surveillance System for Attacks on Health Care^4^).

## Protecting Healthcare

 Last revised in 1949, the Geneva Conventions established explicit protections for healthcare workers (HCWs), medical facilities, and casualties. Articles 19 and 35 of the First Geneva Convention prohibit attacks on medical units and transport, while Article 24 mandates respecting and protecting medical personnel. The Fourth Geneva Convention extends protections to non-combatants, most notably with Article 18 affirming that civilian hospitals must not be the object of attack under any circumstances.

 Sadly, the deliberate targeting of HCWs by combatants has not been new. In their review, Onvlee et al cited examples in Syria, Mosul (Iraq), and others to reiterate the continuation of this dire scenario. On a more positive note, they also quoted examples of adjustments in the delivery of healthcare for adaptation, such as placing hospitals underground to reduce risks of targeting; establishing field hospitals in various locations, including homes, schools, basements, mosques, and caves, before transport to permanent facilities; and reducing working hours so that HCWs could avoid travelling home at night. Community trust in HCWs was a significant factor in ensuring access to care.^1^

 Despite multiple resolutions from the United Nations (UN) Security Council and declarations by member states condemning violence against healthcare, there have been limited trials and involved parties are rarely held accountable. The Safeguarding Health in Conflict Coalition has established an international alliance of governmental and non-governmental organizations to apply diplomatic pressure, and to facilitate data sharing and pool resources. Its recommendations include rejecting any reinterpretation of international humanitarian law (IHL) that would legitimise attacks on healthcare, ending impunity for perpetrators, urging all UN member states to ratify and enforce relevant provisions of international law, expanding data collection, and strengthening leadership and accountability mechanisms at all levels.^6^

 Attacks on HCWs in CAS have severe consequences for local populations. During the Syrian conflict, up to 70% of HCWs have fled the country, and less than two-thirds of hospitals and just over half of primary healthcare centers remain operational.^7^ This has left millions of Syrians without access to essential medical care, contributing to outbreaks of infectious diseases such as polio, measles, and tuberculosis. By 2015, 5.6 million children required assistance, and 24% of pregnant women were adolescents, with the majority lacking access to antenatal and postnatal care. Mental health has also been severely affected, with studies showing that 36%–61% of Syrians exhibit symptoms of post-traumatic stress disorder, and 44% are likely to have a severe mental disorder. The collapse of the healthcare system has resulted in untreated chronic conditions, worsening maternal and child health, and increased morbidity and mortality among local populations. A similar pattern of devastation is now seen in Gaza, where more than 1500 HCWs have been killed and 94% of hospitals have been damaged or destroyed amid ongoing hostilities—the highest toll ever recorded for medical personnel in a single conflict.^8^ At least 772 attacks on healthcare have been reported, leaving clinicians to operate without basic supplies, power, or safety, and further threatening the health and survival of the civilian population. These events underscore the catastrophic consequences of violence against healthcare professionals, which not only deprives communities of essential services but also leads to lasting public health crises.

 Legal frameworks, such as the Geneva Conventions discussed above, provide a structure for potential accountability for attacks on HCWs. However, the current legal frameworks permit justifications for attacking HCWs by invoking the “fog of war” and intelligence-based targeting rationales.^9^ Frequently used tactics include denying responsibility, claiming attacks were accidental, or alleging that medical facilities were being used to shield combatants or store weapons. The UN is largely limited to enacting “soft law,” which includes non-legally binding declarations, arrangements, and interpretations of international obligations. Atrocities can be referred to the International Criminal Court or similar tribunals, such as the International Criminal Tribunal for the former Yugoslavia. Yet, attacks on HCWs carry on to this day, with apparent impunity. Continuous education of all stakeholders, including the younger generations, on the existing IHL and the Geneva Conventions, is essential to improve legal frameworks in the future.

## Performance of Healthcare Workers in Conflict-Affected Settings

 Onvlee et al also identified challenges impacting the performance of the limited health workforce remaining in CAS. Significant issues highlighted were the lack of in-service training, supervision, and task divisions. For example, medical training in Syria did not include specialisation in trauma management, intensive care, or emergency medicine. Surgical teams in the Democratic Republic of Congo were found to be at an increased risk of errors due to the lack of supervision for inexperienced trainees. HCWs had to take on tasks and roles they were not trained for, such as having specialised dialysis nurses in Syria take on the roles of renal specialists.¹

 In recent years, the inadequacies in the medical curricula, namely the importance of addressing the needs of patients suffering from injuries from war, have been recognised and are now changing, often with support from the international communities. For example, the Lebanese University Faculty of Medical Sciences has partnered with the International Committee of the Red Cross to provide a module on the clinical management of war casualties for medical students.^10^ These medical students are also placed in rural and community hospitals closer to the areas of combat, so that they are exposed to and familiarised with conflict-related cases and injuries.

 Courses such as the Basic Emergency Care Course by the WHO, the International Committee of the Red Cross, and the International Federation for Emergency Medicine are now delivered in CAS with an increasing focus on conflict-related injuries.^11^ The course has also moved from in-person to a hybrid mode with an online component, and has now been fully delivered virtually. Initiatives like the Canadian Virtual Medical University Initiative provide online medical training curricula for conflict zones and remote areas.^12^ Together, these training resources have the potential to reach healthcare providers in CAS who face issues with the lack of access to training. Furthermore, digital technology and telemedicine have the potential to be scaled up to provide supervision during care delivery in conflicts.

## Long-term Strategic Planning for the Health Workforce

 One of the strengths of the review by Onvlee et al was the use of the health labour market framework to guide its approach. This framework included not only health labour market dynamics, including the balance between healthcare and other sectors, but also the education sector and the availability of the pool of qualified HCWs. This highlighted the impact of the contextual factors in CAS and its longer-term implications for the health workforce’s production, inflows and outflows, maldistribution, and inefficiencies.^1^

 The review emphasised the importance of longer-term human resources for health policy development for governments and development partners. Similarities can be drawn from our Delphi study on health emergency and disaster risk management workforce development strategies.^13^ In our recommendation for health workforce development policy, experts overwhelmingly agreed that longer-term strategic planning for developing and sustaining the entire health workforce is essential. Specifically, understanding the national labour market dynamics through regular capacity and gap assessments and the development of comprehensive health workforce policies and strategies were ranked amongst the highest importance of the consensus statements.

 Finally, due to the unique and higher demand for HCWs in CAS, the quality of healthcare education has a high impact on the quality of services that can be delivered. Strengthening teachers and trainers, as well as redeveloping curricula, are vital to re-establishing teaching and training standards. Celletti and colleagues suggested that collaboration between the health and education sectors to determine how students are recruited, educated, and deployed has an impact on population health outcomes in low- and middle-income countries.^14^ This approach should also be considered for the long-term development of healthcare in CAS. In addition, with the accessibility of global digital platforms and valuable field research evidence generated in recent years, it remains to be seen what the optimal methods are to effectively update, evolve and sustain frontline medical personnel’s clinical knowledge and public health practices among the mosaic HCW workforces in conflict contexts.^15^

 In the period since the completion of Onvlee and colleagues’ scoping study in August 2022, further conflicts have intensified, the nature of which have not been seen since the end of the Second World War 80 years ago. The very intense and heavy-weapon-concentrated conflicts in Gaza and Ukraine, in particular, have been incredibly destructive for HCWs in those conflicts. The rising trend of conflict-related deaths, the repeated attacks on healthcare facilities, and the repeated violations of IHL seen in recent conflicts are not only worrying, but also deserve our continued and renewed collective attention and efforts in terms of further action and research. Strengthening the accountability mechanism to uphold the IHL, improving data collection and research on health systems in CAS, and enhancing longer-term workforce planning are essential steps towards protecting our health workers and the most vulnerable populations. We must do more to support our HCWs in conflict settings both immediately and in the longer term.

## Disclosure of artificial intelligence (AI) use

 Not applicable.

## Ethical issues

 Not applicable.

## Conflicts of interest

 Authors declare that they have no conflicts of interest.
